# A Room Temperature Ultrasensitive Magnetoelectric Susceptometer for Quantitative Tissue Iron Detection

**DOI:** 10.1038/srep29740

**Published:** 2016-07-28

**Authors:** Hao Xi, Xiaoshi Qian, Meng-Chien Lu, Lei Mei, Sebastian Rupprecht, Qing X. Yang, Q. M. Zhang

**Affiliations:** 1 Department of Electrical Engineering and Materials Research Institute, The Pennsylvania State University, University Park, PA, 16802, USA; 2 Department of Radiology, Penn State College of Medicine, Hershey, PA 17033, USA; 3 Departments of Radiology and Neurosurgery, Penn State College of Medicine, Hershey, PA 17033, USA

## Abstract

Iron is a trace mineral that plays a vital role in the human body. However, absorbing and accumulating excessive iron in body organs (iron overload) can damage or even destroy an organ. Even after many decades of research, progress on the development of noninvasive and low-cost tissue iron detection methods is very limited. Here we report a recent advance in a room-temperature ultrasensitive biomagnetic susceptometer for quantitative tissue iron detection. The biomagnetic susceptometer exploits recent advances in the magnetoelectric (ME) composite sensors that exhibit an ultrahigh AC magnetic sensitivity under the presence of a strong DC magnetic field. The first order gradiometer based on piezoelectric and magnetostrictive laminate (ME composite) structure shows an equivalent magnetic noise of 0.99 nT/rt Hz at 1 Hz in the presence of a DC magnetic field of 0.1 Tesla and a great common mode noise rejection ability. A prototype magnetoelectric liver susceptometry has been demonstrated with liver phantoms. The results indicate its output signals to be linearly responsive to iron concentrations from normal iron dose (0.05 mg _Fe_/g _liver phantom_) to 5 mg _Fe_/g _liver phantom_ iron overload (100X overdose). The results here open up many innovative possibilities for compact-size, portable, cost-affordable, and room-temperature operated medical systems for quantitative determinations of tissue iron.

Iron is a trace mineral that plays a vital role in the human body. Over the past two decades, remarkable progress has been made on the details of iron absorption mechanisms^1^,^2^,^3^. And it has been concluded that absorbing and accumulating excessive iron in body organs (iron overload) can damage or even destroy an organ, such as the liver^4^,^5^,^6^,^7^. The concentration and total amount of iron in different tissues are critical parameters that determine clinical outcome in all forms of iron overload that can be caused by hereditary hemochromatosis, thalassemia intermedia, iron-loading anemias, thalassemia major, sickle cell disease, aplastic anemia, and other pathological disorders or genetic mutations^8^,^9^,^10^,^11^,^12^,^13^,^14^,^15^,^16^,^17^. According to the Centers for Disease Control and Prevention (CDC), even hereditary hemochromatosis alone affects as many as 1 in every 200 people in the U.S. Therefore, it is critical to develop effective means for early and quantitative iron overload detection^18^.

The reference method for evaluating the extent of body iron overload is the measurement of the liver iron concentration (LIC), as liver iron correlates closely with the total body iron. Liver biopsy provides a quantitative measure of iron status and is, therefore, currently considered the “golden standard” for assessing LIC. However, liver biopsy should not be recommended as a routine method due to its invasiveness, discomfort, and significant risk to patients^19^. Among various noninvasive methods, Biomagnetic Liver Susceptometry (BLS) provides a direct method of determining the liver iron concentration *in vivo*, and it has been calibrated and used clinically in studies of patients suffering from iron overload disorders^20^,^21^,^22^,^23^,^24^. With biomagnetic susceptometry, a small magnetic perturbation (response) is induced when a tissue with given magnetic susceptibility is exposed to an external magnetic field (DC bias field). This response can be exploited for diagnostic purposes because both the magnitude and direction of the induced magnetic field vary significantly by tissue types and tissue status with different magnetic susceptibility^25^. On one hand, the response from most human tissues, in which molecules do not contain net magnetic dipoles, is diamagnetic, that is, opposing to the direction of the applied magnetic field, and much weaker in magnitude (~10^−6^). Most non-heme iron atoms in the tissue, often found in ferritin, a ubiquitous intracellular protein, on the other hand, possess net magnetic moments, therefore, it will align with the applied magnetic field and generate a paramagnetic response. In human tissues, this response is directly proportional to the iron atoms present in the tissue. The net biomagnetic response from human tissues, hence, is a linear superposition of these effects^26^,^27^.

In order to meet the required sensitivity and dynamic range detecting the weak AC biomagnetic response^23^,^24^,^25^,^26^,^27^,^28^, the current Biomagnetic Liver Susceptometry (BLS) instrument is based on the Superconducting Quantum Interference Device (SQUID). However, since SQUID magnetometer needs liquid-helium for cryogenic purposes, requires high cost (~$1 million per instrument) and operates with complicated procedures, its accessibility and adoption for general clinical use are significantly limited^27^.

To address the serious technical challenges and vast clinical needs, we have developed a state-of-the-art room temperature susceptometer, utilizing novel magnetoelectric (ME) composites. Our prototype device maintains an ultrahigh sensitivity of ME sensors to detect weak AC biomagnetic signals and introduces a low equivalent magnetic noise of 0.99 nT/rt Hz at 1 Hz under the presence of a strong DC magnetic field (~0.1 Tesla). The results, from investigations on liver tissue mimicking samples with assorted iron concentrations (known as liver phantoms), indicate the output signals to be linearly responsive to iron concentrations from normal (0.05 mg _Fe_/g _liver phantom_) to 5 mg _Fe_/g _liver phantom_ iron overload level.

It has been demonstrated that besides the high AC sensitivity our device also achieved drastic reductions in both its size (<300 cm^3^ for a whole device) and costs. With these improvements, our device will be able to offer a greater portability with minimal maintenance, paving the way to the doctor’s office and bed-side in-patient devices. Considering the wide presence of biomagnetic signals in human organs, the potential impact of such biomagnetic devices on medicine and health care cost could be enormous and far-reaching.

## Results

### High sensitivity to AC biomedical responses from liver phantoms

**Figure 1:**
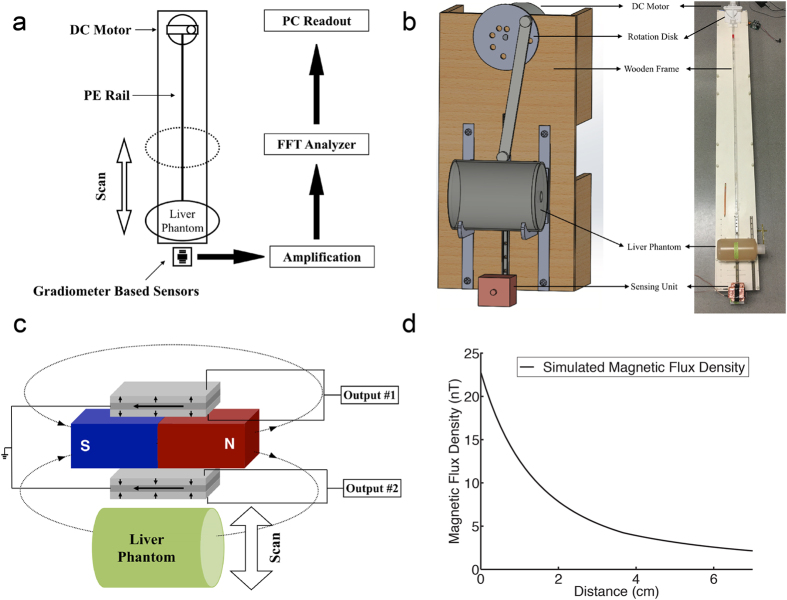
The schematic of the ME-sensor based biomagnetic liver susceptometry (BLS) system. (**a**) The schematic of the operating principle of the ME-sensor based BLS system. FFT Analyzer: Fast Fourier Transform analyzer, PC: Personal Computer, PE Rail: Polyethylene rail. (**b**) The illustration and optical image of the ME-sensor based BLS with a motorized translation stage and the liver phantom. (**c**) The schematic of the working principle of the ME-sensor based first order gradiometer BLS. (**d**) The simulated magnetic flux density from the liver phantom induced by the permanent magnet versus the distance from the liver phantom.

**Figure 2:**
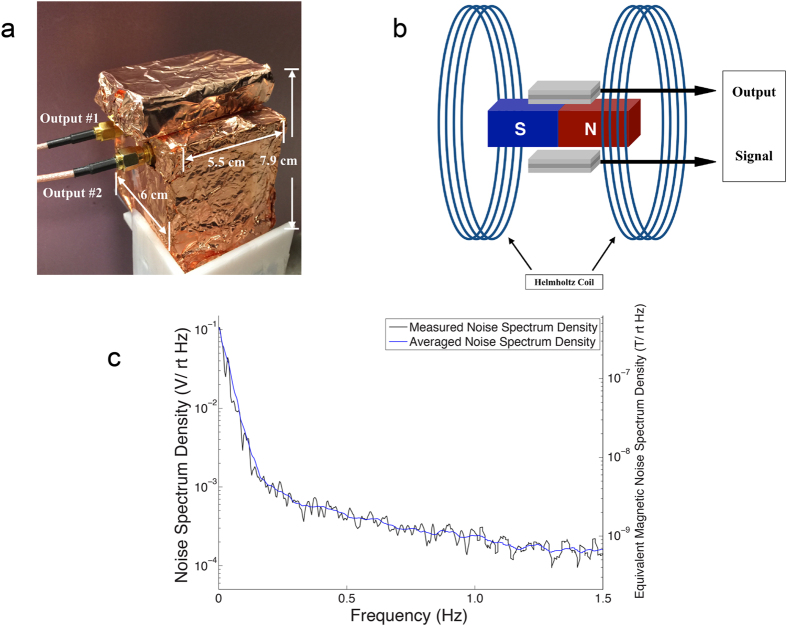
The packaged ME sensor-based BLS system integrated with electronics and sensors. The calibration system for the first order gradiometer. (**a**) An optical image of the packaged ME-sensor based BLS and its dimensions (battery package is also included), shielded by copper foils. (**b**) The schematic of the sensor calibration system for the first order gradiometer. (**c**) The directly acquired noise spectrum from the ME-sensor based BLS (the signal from the ME-sensor based BLS of Fig. 2a was further amplified by 400X).

The prototype of the ME-sensor-based, room-temperature susceptometer system is schematically presented in Fig. 1. It consists of four main compartments (shown in Fig. 1a) – a first order gradiometer sensing unit (shown in Fig. 2a, the optical image and the dimensions of the sensing unit), a set of signal processing instruments for amplification and frequency analysis, a motorized translational motion unit (shown in Fig. 1b) and a personal computer for controlling operation and data acquisition. In the sensing unit, two ME sensors with the same dimension (13 mm × 6 mm × 3 mm) are placed along the direction of the DC magnetic bias field by an N54 Neodymium permanent magnet (2.54 cm × 2.54 cm × 5.08 cm) as illustrated in Fig. 1c. The permanent magnet provides an external DC magnetic field (~30–50 mT) applied to the liver phantom, which, in turn, will be magnetized and then respond with a weak induced field (~10^−6^ of the applied field in magnitude), known as the biomagnetic signal. The biomagnetic signal generated from the iron component in the liver phantom will decay with respect to the separation between the liver phantom and the sensing unit. To demonstrate the relationship that biomagnetic field decreases in the manner of 1/r^3^, we performed computer modeling. Shown in Fig. 1d, where the distance is equal to 0 is the point when the liver phantom surface and device surface touch each other. Liver iron concentration of 0.05 mg _Fe_/g _liver phantom_ is used in the calculation and the liver model has an elliptical shape with three main axes of a = 6 cm, b = 7 cm, and c = 3.5 cm^29^. As shown in Fig. 1d, the magnetic flux densities at two sensor locations are different, and it forms a magnetic field gradient. Our device takes advantage over the magnetic field gradient, as will be discussed with details in the later section.

The motorized stage, as shown in Fig. 1a, is built to scan the phantom at a fixed frequency of 0.5 Hz such that the magnitude of the external DC magnetic field applied to the phantom varies periodically to generate an AC magnetic signal. The moving frequency of the phantom is tuned and monitored by a mechanical switch sensor. The output signal, after amplification, is processed and measured by the Agilent 35670A dynamic signal analyzer.

### Low noise feature of common mode background noise rejection ability

The first order gradiometer measures the gradient of the induced biomagnetic field. It is designed to eliminate common mode environment noises such as the background magnetic noises and temperature fluctuations based on the local gradient characteristics^29^,^30^,^31^,^32^,^33^. The first order gradiometer that we are proposing here employs piezoelectric layers laminated with a magnetostrictive layer (known as the magnetoelectric composite, or the ME composite, refer to Fig. S1 for its operation principles), in a symmetric bimorph configuration. More specifically, Terfenol-D plate (ETREMA Products, Inc. Ames, IA) is chosen as the magnetostrictive layer sandwiched between two piezoelectric PZT ceramic plates (APC International, Ltd. Mackeyville, PA) in each ME sensor. Figure S2 presents the magnetoelectric coefficient of the ME composite sensor. In this piezoelectric arrangement as illustrated in Fig. S3^30^, vibration noises which may lead to bending of the magnetic sensor will not generate electric signal output due to the signal cancellation caused by strain/stress of opposite signs from the top and bottom piezoelectric layers.

In order for the first order gradiometer to operate properly, the two ME sensors in the first-order gradiometer are also expected to be carefully fabricated so that they have an identical ME performance (being close in ME coefficients at an equal DC bias field). Here, the two individual ME sensors were characterized (by standardizing the sensor fabrication process and varying the sensor areas) in the way that under the same circumstances, the outputs from the two ME sensors were nearly the same. Figure 2b is a schematic of the measurement and sensor calibration system. The gradiometer was placed in a fixed position inside a set of Helmholtz coil, which generates a uniform AC magnetic field to both sensors. Then, the amplitudes and phases from the two individual sensors can be recorded by a lock-in amplifier (Stanford Research SR830) for calibration purposes. In our gradiometer design, the difference between the two sensors can be further reduced by adjusting the feedback capacitances of the charge amplifiers, which were directly integrated into the sensor set-up (see Fig. S1 and Eq. (S1)). After these tunings of the two sensors, the difference in the outputs from the two sensor systems (including the charge amplifiers) is less than 1.5 × 10^−5^ V, out of the amplitude of the output signals of 3.51 × 10^−2^ V from each single sensor (under an AC magnetic field of 0.54 Oe). The N54 Neodymium Magnet (2.54 cm × 2.54 cm × 5.08 cm) was fixed in a customized plastic holder, designed to provide the two sensors with an equal DC bias field so that they would be equally sensitive to common mode noises. Customized charge amplifiers were integrated into the sensor system. Copper foil was employed as the shielding enclosure for the gradiometer unit in order to reduce the electromagnetic interferences (EMI), as shown in Fig. 2a.

As stated earlier, when doing liver phantom measurements, the difference in the induced magnetic flux densities at two sensor locations forms a magnetic field gradient, (shown in Fig. 1d). This gradient is detected by the first order gradiometer while the background environmental and temperature noises, which can be considered equal at the two sensors, are eliminated due to the common mode rejection ability of the gradiometer configuration^34^.

The equivalent magnetic noise measurement of the first order gradiometer thus fabricated was performed. Here, the electric output from the first order gradiometer was further amplified via a Stanford Research SR560 differential voltage amplifier by 400X. Figure 2c is the noise spectrum of the gradiometer with electric amplifier circuits, showing the gradiometer has a voltage noise of 0.237 mV/rt Hz at 1 Hz. Using the ME coefficient of Fig. S2 and taking into consideration of 400X signal amplification, it translates into an equivalent magnetic noise of 0.99 nT/rt Hz at 1 Hz under a DC magnetic field of ~0.1 T.

### Linear correlation between liver iron concentrations and system output signals

To characterize the performance of the ME-sensor based BLS, liver phantoms with different iron concentrations were fabricated^35^ and investigated. The phantom recipe can be found in the online supplementary information. The output signals, after amplification, are processed and recorded by the Agilent 35670A dynamic signal analyzer, where the signals are converted into the frequency domain by the Fast Fourier Transformation (FFT) method to examine their amplitude information for each frequency component.

**Figure 3:**
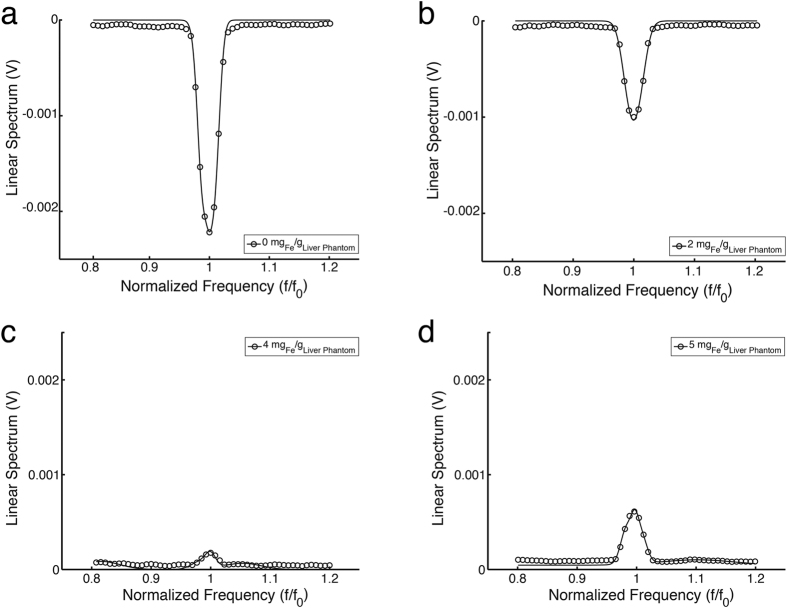
The recorded linear spectrums from liver phantoms with different iron concentrations. (**a**) With 0 mg _Fe_/g _liver phantom_ iron concentration (IC). (**b**) With 2 mg _Fe_/g _liver phantom_ IC. (**c**) With 4 mg _Fe_/g _liver phantom_ IC. (**d**) With 5 mg _Fe_/g _liver phantom_ IC. The recorded spectrum consists of the diamagnetic signal from the water and the paramagnetic response from the irons in the phantom. f_0_ = 0.5 Hz.

**Figure 4:**
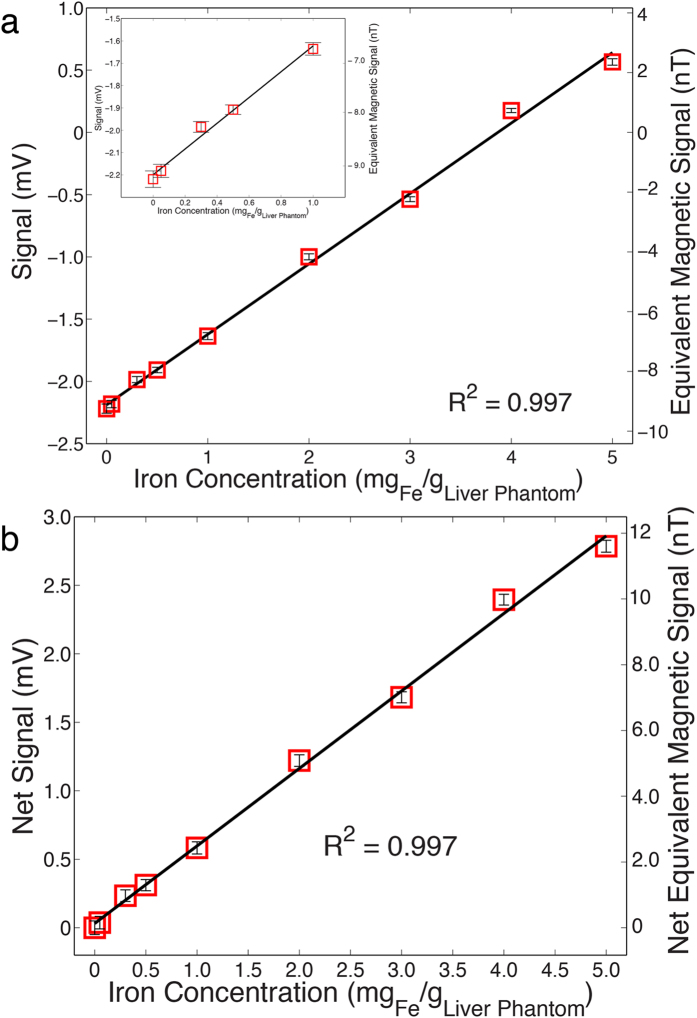
The output signals (amplitude) of the ME-sensor based BLS for a set of liver phantoms with different iron concentrations, ranging from iron normal dose to iron overload. (**a**) Signals of the ME-sensor based BLS for phantoms with iron concentrations from 0 to 5 mg _Fe_/g _liver phantom_. The inset exhibits details, which magnify signals from low iron concentration phantoms (normal LIC). (**b**) The net output signals with respect to the reference, 0 mg _Fe_/g _liver phantom_. Data points and error bars are shown. The solid lines are the linear fitting to the data.

Figure 3 shows the linear spectrums for different iron concentrations from normal iron dose (0.05 mg _Fe_/g _liver phantom_) to 5 mg _Fe_/g _liver phantom_ iron overload (100X overdose) versus its respective normalized frequency (the scanning frequency f_0_ = 0.5 Hz). Figure 4a plots the amplitudes of each peak from a given investigation versus its corresponding iron concentration for every liver phantom. Each data point is the averaged value of multiple stably repeatable measurements. By the linear regression method, an excellent linearity lying between the output signals and their corresponding LICs is found.

Furthermore, since our major interest lies on the susceptibility of the liver phantoms with respect to water (reference), in other words, the amount by which the susceptibility of water is increased as paramagnetic iron is being added, we deducted the water contribution from the overall signal for a better demonstration, resulting in Fig. 4b. An excellent linearity between iron concentration and (net) output signal is observed, with the coefficient of determination being 0.997.

## Discussion

Data in Fig. 3 are consistent with the fact that while water is a major component in the phantoms and shows a diamagnetic susceptibility of −9.396 × 10^−6^ SI (measured with respect to air, namely, χ_w_ − χ_air_)^24^, with increasing iron (paramagnetic) levels, the overall susceptibility of the liver phantom is expected to gradually change over to be paramagnetic, passing through the point when contributions of both water and iron are equal, resulting an effective susceptibility of zero, as shown in Fig. 3c. The results are in good agreement with those from SQUID-based BLS as well as MRI measurements^5^,^6^. Additionally, the inset of Fig. 4a suggests that the ME-sensor-based BLS has the sensitivity for the detection of iron concentrations from 0.05 mg _Fe_/g _liver phantom_ to 0.5 mg _Fe_/g _liver phantom_, a conventional LIC range that is clinically considered as normalcy^36^.

It is noted that the sensitivity of the ME sensor can be improved by 10X via replacing the piezoceramic PZT with piezo-single crystals^37^. Moreover, employing a second order gradiometer as in the SQUID BLS instead of the first order gradiometer used here can lead to an order of magnitude reduction on the noise level^38^. These improvements will enable the ME sensor system to be used in the clinical applications to compensate for more than one order reductions in the magnitude of the liver biomagnetic signal, due to the increased separation between the liver and the sensor system, as well as the diamagnetic signals from the overlaying tissues.

In summary, the LIC level is of extreme importance in determining one’s overall body iron status. We have successfully developed an ME-sensor-based BLS and demonstrated that it can be operated in an un-shielded environment for assessing LIC. Our development of the ME-sensor-based BLS technology is both “conventional” and “disruptive”. It is conventional in the sense that this technology will follow the well-developed methodology of the SQUID-based BLS, which has been proven to be effective in quantifying the LIC over a broad range of iron concentrations. It is disruptive, on the other hand, because the technology can lead to breakthroughs in cost, size and, most importantly, clinical applicability. The ME-sensor-based BLS eliminates most of the limitations in clinical applications associated with the SQUID-based ones, without adding any new problems. We foresee our work to be inspirational and believe that reporting this breakthrough to the public will help it be developed at an even faster pace so that more people can soon benefit from this technology.

## Additional Information

**How to cite this article**: Xi, H. *et al*. A Room Temperature Ultrasensitive Magnetoelectric Susceptometer for Quantitative Tissue Iron Detection. *Sci. Rep.* **6**, 29740; doi: 10.1038/srep29740 (2016).

## Supplementary Material

Supplementary Information
