# Supplementary material for: A Room Temperature Ultrasensitive Magnetoelectric Susceptometer for Quantitative Tissue Iron Detection
Source: Sci Rep. 2016 Jul 28;6:29740. doi: 10.1038/srep29740 (PMC4964576; doi:10.1038/srep29740)
Supplement: Supplementary Information [file srep29740-s1.pdf]

Online Supplementary Information

A Room Temperature Ultrasensitive Magnetoelectric Susceptometer for Quantitative Tissue Iron Detection

Hao Xi[1*], Xiaoshi Qian[1,*a], Meng-Chien Lu[1], Lei Mei[1], Sebastian Rupprecht [3], Qing X Yang[2], and Q. M. Zhang[1,a]

[1]Department of Electrical Engineering and Materials Research Institute, The Pennsylvania State University, University Park, PA, 16802, U.S.A.

[2]Departments of Radiology and Neurosurgery, Penn State College of Medicine, Hershey, PA 17033, U.S.A.

[3]Department of Radiology, Penn State College of Medicine, Hershey, PA 17033, U.S.A.

This file contains:

Method

Supplementary Figures

Fig S1-S3

Method

ME sensors

ME laminate composites are a recent development that has the promise for compact-size, low-cost and room-temperature-operated biomagnetic liver susceptometry with an ultrahigh sensitivity [39-45]. By employing magnetoelectric composite principles, the sensitivity of the ME sensor system in Fig. 2 can be expressed as[46-49],

$$\frac{V_{out}}{\Delta \mathrm{H_a}} \approx \frac{\Delta \mathrm{q}_p}{\mathrm{C_f}\Delta \mathrm{H_a}} = \frac{d_p A_p}{\mathrm{C_f} s_p^E \left(1+\frac{s_m^H t_p}{s_p^E t_m}\right)} \frac{\Delta S}{\Delta \mathrm{H_a}} \qquad \text{(S1)}$$

where$\triangle S/\triangle H_a$ is the piezomagnetic coefficient in the magnetostrictive layer, d_p is the piezocoefficient and A_p is the total electrode area of the piezo-layer, t_p, t_m, s_p^E and s_m^H are the thicknesses and the compliances of piezo-layer and magnetic layer respectively, q_p is the charge generated in the piezo-layer[50, 51]. Equation (S1) shows the dependence of the field sensitivity of the ME sensor on the material parameters such as piezo-coefficient d_p, magnetostrictive coefficient $\Delta S_m/\Delta H_a$, and elastic compliance of both the piezo- and magneto-layers. The PZT/Terfenol-D sensor, with high piezo-coefficient and magnetostrictive coefficient, which has an equivalent magnetic noise of 0.99 nT/rt Hz at 1 Hz, shows promise for applications on the biomagnetic sensors. Fig. S3 is the ME coefficient of PZT/Terfenol-D sensors which has a maximum coefficient around 0.6 V/cmOe. As shown in Fig. S4, after 400X amplification, at 0.5 Hz, the noise level in Channel A is 185 μV. After the first order gradiometer, the system noise level is reduced to 50.8 μV at 0.5 Hz. Note that for the separate noise measurement of each channel, a low-frequency noise at approximately 1.5 Hz was observed. As expected, due to the common mode noise rejection ability of the first order gradiometer configuration, it is eliminated.

The Terfenol-D (ETREMA Products, Inc. Ames, IA) plates with a dimension of 13.0 × 6.0 × 1 mm^3 are aligned preferentially in the length direction. The PZT plates (APC International, Ltd.Mackeyville, PA) have dimensions of 13 × 6.0 × 1 mm^3 with the top and bottom surface electrodes fired with silver and poled in the thickness direction. The PZT and Terfenol-D are bonded by low-viscosity epoxy EP5340 (Eager Polymers, Chicago, IL) with an antiparallel electric connection.

Liver Phantom Preparation

Tissue-mimicking materials are adopted to customize the design and fabrication of the liver phantoms. $FeCl_2$ (Iron II chloride) (Sigma 372870) was used for the liver iron; the solution is mixed with Agar (Sigma A7921) and heated until boiling. DI water (dH_20) is mixed with Agar (Sigma A7921) into the beaker of 1500 ml with a proper ratio. The mixed solution is then de-gassed by heating slowly to boil with magnetic stirs in it. $FeCl_2$ is added to the boiled Agar/Water solution after it cools down below 70 ^{o}C.

After the solution is made, it is slowly poured into the phantom container (preventing extensive bubbling). The rest of DI water is added till the bottle filled up. The container is carefully shaken to get bubbles rise up to its mouth. The bubbles are then removed with a syringe.

References:

39. Li, F., Zhao, F., Zhang, Q. M. & Datta, S. Low-frequency voltage mode sensing of magnetoelectric sensor in package. *Electron. Lett.*. **46**, 1132-1134, (2010).
40. Lu, M.-C., Mei, L., Jeong, D.-Y., Xiang, J., Xie, H. & Zhang, Q. M. Enhancing the magnetoelectric response of Terfenol-D/polyvinylidenefluoride/Terfenol-D laminates by exploiting the shear mode effect. *Appl. Phys. Lett*. **106**, 112905, (2015).
41. Lage, E., et al. Exchange biasing of magnetoelectric composites. *Nature Mater*. **11**, 523-529 (2012).
42. Li, F., Misra, R., Fang, Z., Wu, Y., Schiffer, P., Zhang, Q. & Datta, S. Magnetoelectricflexural gate transistor with nanotesla sensitivity. *J. Microelectromechanical Sys*t. **22**, 71-79, (2013).
43. Ramesh, R., Spaldin, N. A., "Multiferroics: progress and prospects in thin films," *Nature Mater*. **6**, 21–29 (2007).
44. Fang, Z. Lu, S. G., Li, F., Datta, S., Zhang, Q. M. & Tahchi, M. El. Enhancing the magnetoelectric response of Metglas/polyvinylidene fluoride laminates by exploiting the flux concentration effect. *Appl. Phys. Lett.* **95**, 112903, (2009).
45. Gillette, S. M., Geiler, A. L., Gray, D., Viehland, D., Vittoria, C. & Harris, V. G. Improved sensitivity and noise in magneto-electric magnetic field sensors by use of modulated AC magnetostriction. *IEEE Magn. Lett*. **2**, 2500104, (2011).
46. Eerenstein, W., Mathur, N. D., & Scott, J. F., Multiferroic and Magnetoelectric Materials. *Nature*, 442, 759-765 (2006).
47. Dong, S. X., Li, J. F. & Viehland, D. Characterization of magnetoelectric laminate

composites operated in longitudinal-transverse and transverse–transverse modes. *J. Appl. Phys.* **95**, 2625–2630, (2004).

48. Mei, L., Rupprecht, S., Yang, Q. & Zhang, Q. M. A first order gradiometer based low noise magnetoelectric sensor system. 71st Device Research Conference (DRC), 85-86, (2013).

49. Fang, Z., Mokhariwale, N., Li, F., Datta, S. & Zhang, Q. M. Magnetoelectric sensors with directly integrated charge sensitive readout circuit—Improved field sensitivity and signal-to-noise ratio. *IEEE Sens. J.* **11**, 2260-2265, (2011).

50. Nan, Ce-Wen, et al. Multiferroicmagnetoelectric composites: historical perspective, status, and future directions. *J. Appl. Phys.* **103,** 031101, (2008).

51. Mori, Kiyotake, & Wuttig, Manfred. Magnetoelectric coupling in terfenol-D/polyvinylidenedifluoride composites. *Appl. Phys. Lett.* **81**,.100-101, (2002).

Fig.S1.

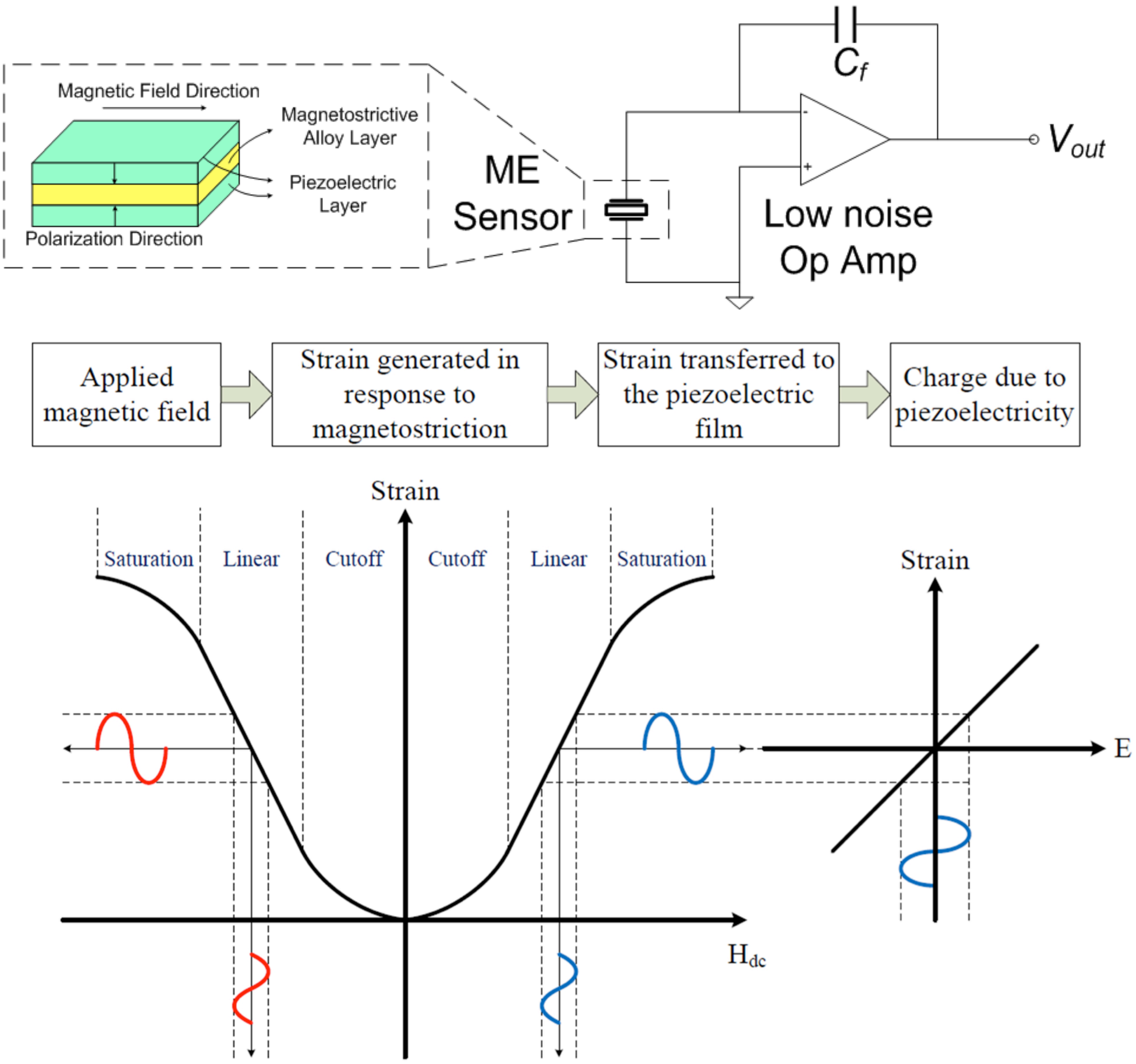

Fig. S1. (top) The schematic of an ME sensor system consisting of the ME laminate and the readout electronics. (bottom) the schematic illustration of the operating principles of an ME magnetic sensor. By tailoring the magnetostrictive layer composition, the ME sensor can have a high sensitivity to detect the weak AC biomagnetic signal while under a strong external DC magnetic field.

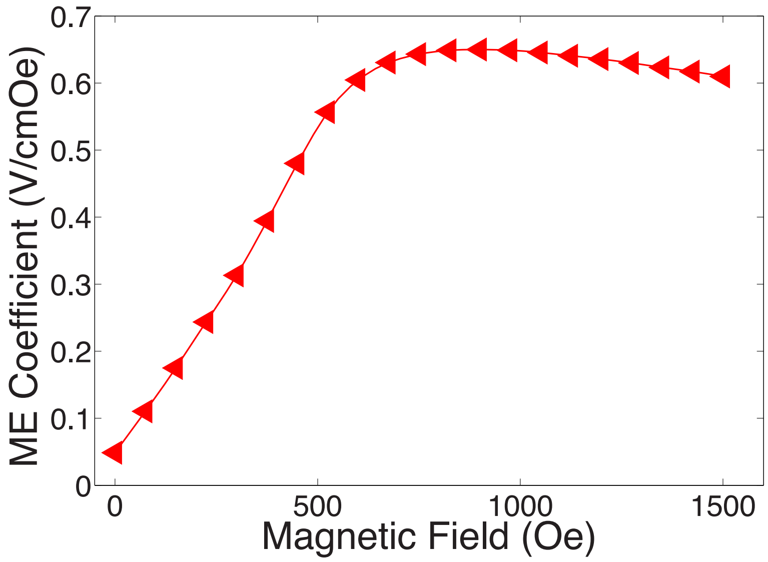

Fig. S2. The ME coefficient as a function of magnetic bias field for PZT/Terfenol-D/PZT composite sensor. The thickness of the PZT plates and Terfenol-D is 1 mm and the total sensor thickness is 3 mm.

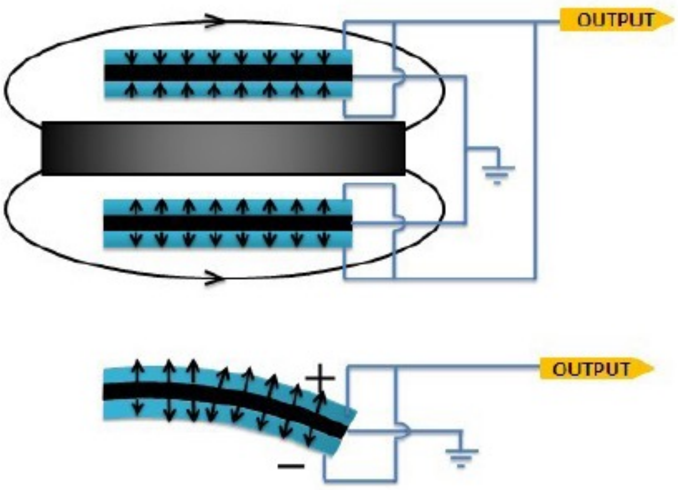

Fig. S3. (Top) A first order gradiometer and (Bottom) the illustration of how the two piezo-layers in an ME sensor cancel the vibrational noise (induced bending). The piezoelectric poling-direction in each piezo-layer is indicated.

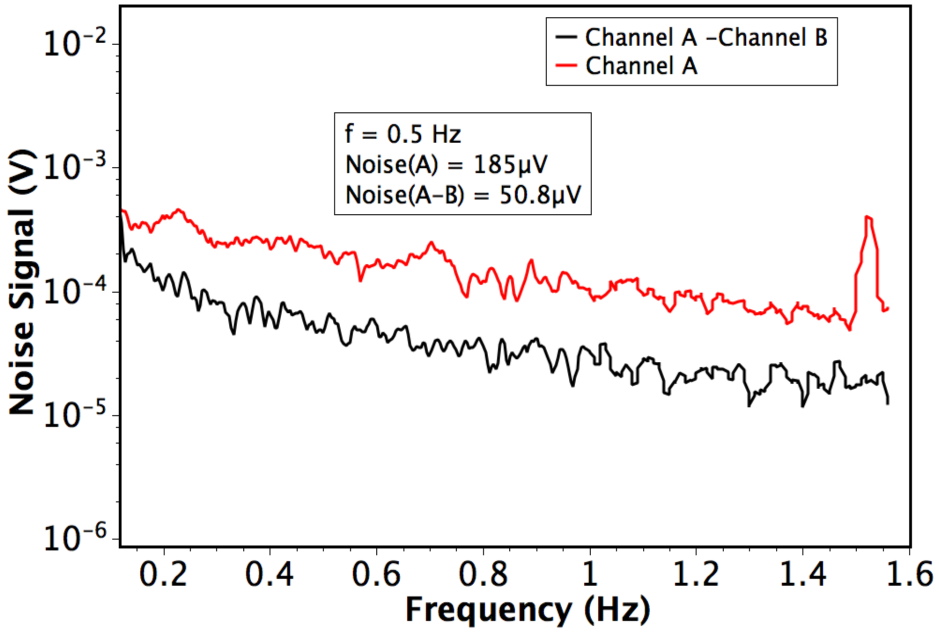

Fig. S4. The noise spectra of the sensor system. Noise(A) is from a single sensor and Noise(A-B) is from the first order gradiometer. Note that Noise(A) has a noise peak from an unknown source at approximately 1.5 Hz. This noise peak is eliminated due to the common mode rejection ability of the first order gradiometer configuration.
